# Raw Lacquer Extract from *Toxicodendron vernicifluum* in Combination with ONC201 Enhances the Inhibitory Effects on Colorectal Cancer Cell Activity

**DOI:** 10.5152/tjg.2022.22048

**Published:** 2023-03-01

**Authors:** Zhezhu Jin, Yongjun Jin

**Affiliations:** 1 Department of Colorectal Surgery, Hangzhou Red Cross Hospital, Hangzhou Zhejiang, China

**Keywords:** Colorectal cancer, mTOR, ONC201, raw lacquer extract, Toxicodendron vernicifluum (stokes)

## Abstract

**Background:**

The purpose of the present research was to explore the therapeutic impact of raw lacquer extract from *Toxicodendron vernicifluum* on colorectal cancer cells and to investigate the outcome of raw lacquer extract and ONC201 co-treatment on the activity of colorectal cancer cells.

**Methods:**

The cells of HCT116 were treated with raw lacquer extract, ONC201, or co-treatment. Subsequently, MTT, trypan blue staining, colony formation, annexin V/propidium iodide staining, wound healing, and transwell assays were performed to assess the effects of raw lacquer extract, ONC201 and the synthesis effect of co-treatment on cell activity, survival, proliferation, apoptosis, migration, and invasion in HCT116 cells. Western blotting and immunostaining assay were also performed to detect the expressions of tumor necrosis factor-related apoptosis-inducing ligand, death receptor-5, cleaved caspase-8, p-mTOR/mTor, and p-S6K/S6K in cells.

**Results:**

The results showed that ONC201 and raw lacquer extract had effective anti-cancer effects on HCT116 cells. ONC201 and raw lacquer extract treatment on colorectal cancer cells inhibited cell viability and growth, as well as induced cell apoptosis and cell death of HCT116. The migration and invasion of HCT116 cells were also inhibited. Significantly, raw lacquer extract and ONC201 co-treatment further enhanced the anti-colorectal cancer cell activity in HCT116 cells. Western blotting and immunostaining assay showed that raw lacquer extract in combination with ONC201 induced tumor necrosis factor-related apoptosis-inducing ligand/death receptor-5 expression activation, inhibited the expression of cleaved caspase-8/procaspase-8, and reduced the expression of p-mTOR/mTOR and p-S6K/S6K.

**Conclusion:**

These results indicated that raw lacquer extract in combination with ONC201 enhanced the inhibitory effects on colorectal cancer cell activity.

**Main Points**

- ONC201 and RLE had effective anti-cancer effects on colorectal cancer (CRC) cells.
- RLE in combination with ONC201 enhanced the inhibitory effects on CRC cell activity.
- RLE in combination with ONC201 induced TRAIL/DR-5 expression activation, inhibited cleaved caspase-8/procaspase-8, p-mTOR/mTOR, and p-S6K/S6K.

## Introduction

Colorectal cancer (CRC) was graded as the third most common human malignant cancer worldwide, had a poor prognosis, and a high mortality rate in both genders of the adult population. The report of global cancer statistics shows that the new cases of CRC were over 1.8 million and the deaths were estimated at 881 000 in 2018, amounting to 1/10 of all cancer cases and deaths.^1^ Currently, CRC screening, surgical management, adjuvant chemotherapy, and radiotherapy are the main strategies for the management of CRC and are used to provide effective improvement in patients’ life quality and 5-year survival rate.^2,3^ However, tumor invasion, metastasis, and recurrences still prevent CRC patients from recovering and reduce the long-term survival rate. Therefore, developing effective and safe therapeutic agents is urgent to improve the prognosis and survival outcomes of patients with CRC.

The death ligand, tumor necrosis factor-related apoptosis-inducing ligand (TRAIL), has become an interesting therapeutic target for the treatment of CRC and other human malignant tumors.^4^ ONC201, a small molecular inducer of TRAIL, has been developed with the ability to regulate TRAIL and death receptor-5 (DR5) which is the TRAIL’s receptor.^5^ Previous scientist’s research has shown that ONC201 has effects on proliferation and promotes apoptosis of a broad range of tumor cell types, and the underlying biological action of this may be related to the upregulation of TRAIL and forkhead box O3 (Foxo3a), as well as the inhibition of Akt and Erk in tumor cells.^6,7^ A study also found that mTOR block could sensitize ONC201-induced anti-CRC cell survival by blocking mTOR and Akt signaling.^8^ Scientists have used it in phase II clinical trials on a variety of advanced cancers.^6^ However, ONC201 has been found to have a high tolerance in multiple advanced types of human cancers.^9^ Wagner et al^10^ revealed a promising treatment for CRC, which is ONC201 in combination with the anti-angiogenic agent, bevacizumab. This combination treatment acts through a unique mechanism to increase tumor cell death and inhibit cell proliferation in vivo. Combined medication is a great therapeutic to improve treatment efficiency, reduce the risk of side effects, and improve the survival of patients.^11^

*Toxicodendron vernicifluum (T. vernicifluum) *(Stokes) F. A. Barkley (syn.* Rhus verniciflua *Stokes, RVS*)* is a kind of deciduous tree and is famous for producing raw lacquer, which is the resin from the bark of this plant. Recently, various biological activities of *T. vernicifluum *have been reported, and most notable are anti-inflammation and anti-bacterial activities.^12^ It has been also utilized as an herbal medicine throughout the history of China, including for abdominal disorders, and the dried raw lacquer of RVS after processing is mainly used to promote blood circulation and stasis.^13^ A Korean single-center study reported that complementary treatment with the standardized extraction of this plant is beneficial for patients with metastatic CRC, with treatment being positively associated with overall survival without significant side effects.^14^ Further studies have also found that the active compound, fisetin, in *T. vernicifluum*, could inhibit human cancer cell growth and reduce the phosphorylation of mTOR.^15,16^

In this research, we hypothesized that raw lacquer extract (RLE) of *T. vernicifluum *has an anti-cancer effect against CRC, by raising the potency of ONC201 treatment for the prevention of CRC. Therefore, the therapeutic effect of RLE in CRC cells was investigated, and the role of RLE on ONC201-induced anti-cancer activity was further explored.

## Materials and Methods

### Preparation of Raw Lacquer Extract

Dried standardized allergen-removed raw lacquer was extracted with 60% methanol at room temperature for 24 hours, with 3 repeats of this step. Then, the liquid was filtered and freeze-dried to obtain the RLE. The obtained RLE was further stored at −80°C for subsequent experiments.

## Cell Culture and Treatment

Two cell lines were used in this experiment: NCM460, a normal colonic epithelial cell line, and HCT116, a CRC cell line, were purchased from iCell Bioscience, Inc., China. Both were fostered in RPMI-1640 medium (Gibco) provided by Thermo Fisher Scientific, Inc., Waltham, Massachusetts, USA, complemented with 10% fetal bovine serum and 1% penicillin–streptomycin solution (5% CO_2_, 37°C, 95% incubator).

### MTT Assay

MTT (3- [4, 5-dimethylthiazol-2-yl]-2, 5-diphenyl tetrazoliumbromide) was used to test the cell survival following RLE and/or ONC201 treatment. NCM460 or HCT116 cells (1 × 10^4^ cells per well) were cultured into 96-well plates, treated with 4 doses of RLE (0.125 mg/mL, 0.25 mg/mL, 0.5 mg/mL, and 1.0 mg/mL) or ONC201 (10 μM) for 24 hours and 48 hours. The dosages of RLE and ONC201 were based on previous studies.^8,17^ Subsequently, 5 mg/mL MTT reagent (M5655, Sigma-Aldrich, Merck KGaA, Darmstadt, Germany) was added to the plates for incubating a further 4 hours at 37°C. The formed crystals were dissolved with Dimethyl sulfoxide (DMSO, R21950, Yuanye, Shanghai, China), and the activity was gauged at 590 nm by a CMaxPius ELISA reader (SpectiaMax, Molecular Devices, Sunnyvale, USA) that was expressed by optical density value.

### Trypan Blue Staining Assay.

To judge the cytotoxicity of RLE on HCT116 cells, 0.4% trypan blue solution was exerted. After the treatment, the trypan blue-positive cells number was counted.

### Cell Colony Formation Assay

HCT116 cells were divided into 5 × 10^2^ cells per well equally. Following treatment, cells were cultured until 14 days and the media was changed every 3 days. At the end of the culturing, the positive cell clusters stained with 0.5% crystal violet were counted by a microscope (AE2000; Motic Deutschland GmbH).

### Annexin V/Propidium Iodide Staining Assay

The degrees of cell apoptosis were detected using the relative staining kit [Hangzhou MultiSciences (Lianke) Biotech Co., Ltd., China] by the annexin V/propidium iodide (PI) staining assay.^18^ Briefly, cells have been placed into 6-well plates until the logarithmic phase and then treated with RLE and/or ONC201 for 48 hours. The cells were digested and resuspended in the 200 μL binding reagents consisting of 10 μL annexin V-FITC and 5 μL PI. Then, they were incubated at room temperature without light for 15 minutes as stated in the manufacturer’s instructions. The cells were immediately detected by a flow cytometer BD Accuri C6 (BD Biosciences, Puerto Rico, USA).

### Wound Healing Assay

The wound healing assays assessed cell migration.^19^ Cells with 5 × 10^5^ per well were seeded into 6-well plates and fostered overnight in a 5% CO_2_ incubator at 37°C. Linear wound tracks were created using sterile pipettes. The cells were then swilled gently with phosphate-buffered saline (PBS) to remove the loose cells and treated with RLE and/or ONC201. The images of the wound healing were photographed at 0 hours, 24 hours, and 48 hours, and the migration rate (%) was also calculated using the National Institutes of Health’s software, Image J.

### Transwell Invasion Assay

Transwell assays were exerted to assess the cell invasive abilities as previously described.^20^ Briefly, HCT116 cells (5 × 10^4^) treated with RLE and/or ONC201 were added to the upper chamber, and the regular medium filled the lower chambers. The chambers were gestated for 24 hours at 37°C until invasion into the lower chamber. After cleaning the upper and lower chambers with PBS once, 100 μL calcein-AM solution was added into the lower chamber and gestated at 37°C for 60 minutes. The cells were observed using an inverted microscope and analyzed with Image J software. The count of invaded cells was then calculated.

### Western Blotting

Cells’ proteins were extracted in Radioimmunoprecipitation assay buffer (RIPA) (P0013E, Beyotime, Shanghai, China) and detected by bicinchoninic acid (BCA) assay (Beyotime, Shanghai, China). Then, samples were separated on 5% sodium dodecyl sulfate–polyacrylamide gel electrophoresis by electrophoresis and transferred onto polyvinylidene difluoride membranes. Following blocking the membranes in 5% non-fat milk for 2 hours at normal atmospheric temperature, the membranes were rocked with TBST 3 times for 10 minutes and then swayed and incubated with the primary antibodies namely anti-TRAIL (ab2056, 1:500, Abcam), anti-DR-5 (ab199357, 1:1000, Abcam, Cambridge, UK), anti-cleaved caspase 8 (number 9496S, 1:1000, CST), anti-pro-caspase 8 (ab108333, 1:5000, Abcam), anti-mTOR (ab32028, 1:2500, Abcam), anti-p-mTOR (ab109268, 1:5000, Abcam), anti-S6K (ab32359, 1:2500, Abcam), anti-p-S6K (ab59208, 1:1000, Abcam), anti-β-actin (ab6276, 1:10000, Abcam) at 4°C overnight. The membranes were then washed with TBST and further incubated with the corresponding secondary antibody for 1 hour at normal atmospheric temperature. The protein bands were observed by an ECL system, and band intensity was measured with ImageJ. β-actin acted as the internal control to standardize the relative protein density.

### Immunofluorescence Assay

The expression profiles of TRAIL, B-cell lymphoma-2 (Bcl-2, Abcam, Cambridge, UK), and Bax in HCT116 cells were determined by immunofluorescence. Briefly, HCT116 cells were fixed with 4% paraformaldehyde after removing the culture medium and then incubated with 0.1% Triton X-100 for 15 minutes. Subsequently, cells were cleaned with PBS 3 times, and 2% bovine serum albumin (BSA) was further added to soak for 1 hour. A further wash with PBS 3 times was then performed. Cells were then covered with primary antibodies: TRAIL (number 3219S, 1:400, CST), Bcl-2 (ab218123, 1:1000, Abcam), and Bax (ab53154, 1:500, Abcam) at 4°C until the next day, then cleaned with PBS 3 times again and further covered with the secondary antibodies and 4’,6-diamidino-2-phenylindole (DAPI, R20274, Shanghai Yuanye, China) for 1 hour at room temperature. Cells were photographed using an inverted fluorescent microscope.

### Statistical Analysis

Experimental data are expressed as the mean ± standard deviation. Statistical analysis was carried out by one-way analysis of variance followed by the post hoc least significant difference (LSD) test for multiple comparisons, which was performed by The Statistical Package for Social Sciences version 25.0 software (IBM Corp.; Armonk, NY, USA).

## Results

### Raw Lacquer Extract Inhibits HCT116 Cell Viability 

**Figure 1.**
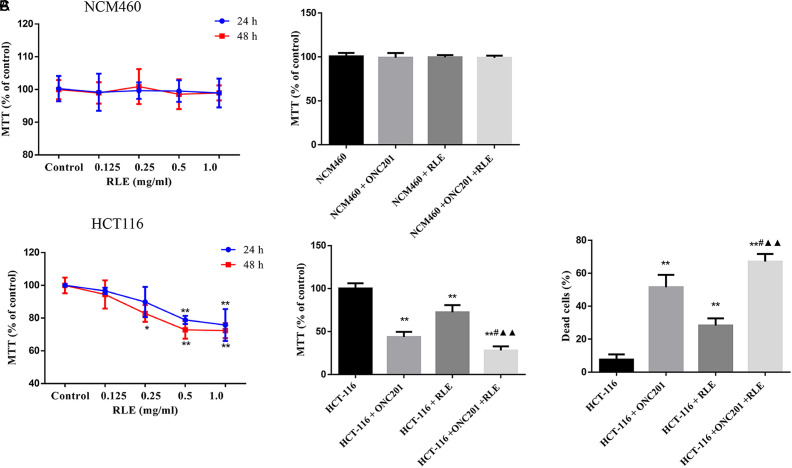
RLE inhibits HCT116 cell viability and strengthens ONC201 induced cell death. (A and B) Cell viability was detected using MTT assay. (C) Cytotoxicity was examined using trypan blue staining assays. ^*^ *P* < .05, ^**^ *P* < .01 vs. the HCT116 group; ^#^ *P* < .05, ^##^ *P* < .01 vs. the HCT116 + ONC201 group; ^▲^ *P* < .05, ^▲▲^ *P* < .01 vs. the HCT116 + RLE group. RLE, raw lacquer extract.

First, normal colonic epithelial cell NCM460 was processed with RLE or ONC201 to assess the impact of RLE (0.125 mg/mL, 0.25 mg/mL, 0.5 mg/mL, or 1.0 mg/mL) and ONC201 on non-tumor cells. The results in Figure 1A showed that RLE and ONC201 treatment did not change the viability of NCM460 cells. Then, HCT116 cells were further handled with the planned consistency of RLE for 24 hours and 48 hours. The results indicated that RLE at 0.5 mg/mL and 1.0 mg/mL had a significant repressive impact on the viability of HCT116 cells for 24 hours and 48 hours of treatment (*P *< .01) (Figure 1B). Comparing the HCT116 cell viability after administration of 0.5 mg/mL and 1.0 mg/mL RLE, it could be found that the effect of inhibiting cell viability is not much improved after doubling the dosage. Therefore, 0.5 mg/mL RLE was picked for succeeding experiments. Further co-treatment with ONC201 (10 μM) and RLE (0.5 mg/mL) showed that ONC201, RLE, and co-treatment significantly inhibited HCT116 cell viability (*P *< .01) (Figure 1B). In addition, ONC201 (10 μM) and RLE (0.5 mg/mL) co-treatment significantly increased the inhibitory effect on HCT116 cell viability compared to the ONC201 or RLE handled alone (*P *< .05 and *P *< .01, respectively).

### Raw Lacquer Extract Strengthens ONC201 Induced Cell Death

The trypan blue assay outcomes in Figure 1C demonstrated that RLE (0.5 mg/mL) or ONC201 (10 μM) treatment both exhibited cytotoxicity on HCT116 cells and induced cell death significantly (*P *< .01). Moreover, ONC201 (10 μM) and RLE (0.5 mg/mL) co-treatment strengthened the ONC201- or RLE-induced cell death significantly (vs. the RLE group, *P *< .05 and vs. the ONC201 group, *P *< .01).

### Raw Lacquer Extract Inhibited HCT116 Cell Proliferation

**Figure 2.**
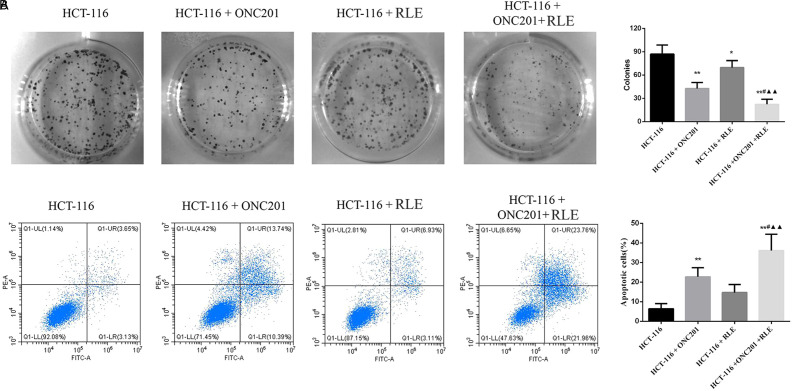
RLE inhibits HCT116 cell growth and facilitates ONC201 induced HCT116 cell apoptosis. (A) Colony formation assays were performed to detect cell growth. (B) Annexin V/PI staining assays were performed to assess cell apoptosis. ^*^ *P* < .05, ^**^ *P* < .01 versus the HCT116 group; ^#^ *P* < .05, ^##^ *P* < .01 versus the HCT116 + ONC201 group; ^▲^ *P* < .05, ^▲▲^ *P* < .01 versus the HCT116 + RLE group. RLE, raw lacquer extract.

We determined the effects of RLE on HCT116 cell growth by cell colony formation assays. The results showed that the cell colony formation numbers in the RLE (0.5 mg/mL) treatment group were reduced after treatment with 0.5 mg/mL RLE for 48 hours and further cultured for 14 days. Similar results were also observed in the ONC201 (10 μM) treatment group and the ONC201+RLE treatment group (Figure 2A). Notably, the RLE (0.5 mg/mL) and ONC201 (10 μM) co-treatment enhanced the inhibitory effect on HCT116 cell proliferation enormously compared with the single treatment groups (*P *< .05 and *P *< .01, respectively).

### Raw Lacquer Extract Facilitates ONC201 Induced HCT116 Cell Apoptosis

To inquire about the impact of RLE on apoptosis of CRC cells, annexin V staining assays were performed (Figure 2B). The results showed that compared with the control HCT116 cell group, the ONC201 (10 μM) treatment significantly induced HCT116 cell apoptosis (*P *< .01). Raw lacquer extract (0.5 mg/mL) and ONC201 (10 μM) co-treatment facilitated the stimulation effect on HCT116 cell apoptosis compared with the individually handled groups (*P *< .05 and *P *< .01, respectively).

### Raw Lacquer Extract and ONC201 Co-Treatment Inhibits HCT116 Cell Migration and Invasion 

**Figure 3.**
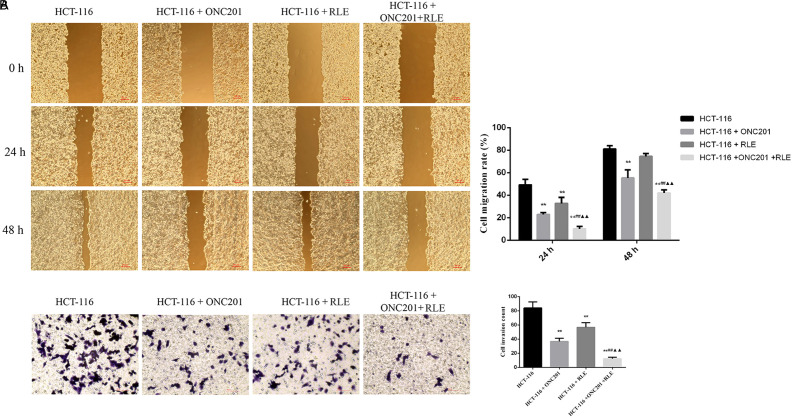
RLE and ONC201 co-treatment inhibited HCT116 cell migration and invasion. (A) Wound healing assays were performed to detect cell migration. (B) Transwell assays were performed to detect cell invasion. ^*^ *P* < .05, ^**^ *P* < .01 versus the HCT116 group; ^#^ *P* < .05, ^##^ *P* < .01 versus the HCT116 + ONC201 group; ^▲^ *P* < .05, ^▲▲^ *P* < .01 versus the HCT116 + RLE group. RLE, raw lacquer extract.

The effects of RLE (0.5 mg/mL) and ONC201 (10 μM) co-treatment on HCT116 cell migration and invasion were studied by the wound healing and transwell assays (Figure 3). As the Figure 3A indicated, ONC201 (10 μM) as well as ONC201 (10 μM) and RLE (0.5 mg/mL) co-treatment all inhibited HCT116 cell migration significantly at 24 hours and 48 hours after scratching (all *P *< .01); RLE (0.5 mg/mL) treatment inhibited HCT116 cell migration significantly at 24 hours after scratching (*P *< .01). Furthermore, the cell migration rate in the ONC201 (10 μM) and RLE (0.5 mg/mL) co-treatment group was further reduced in comparison to that in the individual treatment groups at 24 hours and 48 hours after scratching, respectively (all *P *< .01). As shown in Figure 3B, transwell assay showed that ONC201 (10 μM), RLE (0.5 mg/mL), and co-treatment reduced the number of invaded HCT116 cells substantially (*P *< .01, *P *< .05, and *P *< .01, severally), and the number of invaded cells in the ONC201 (10 μM) and RLE (0.5 mg/mL) co-processing group was remarkably lower in comparison to the ONC201 (10 μM) and RLE (0.5 mg/mL) individual treatment groups (*P *< .01 for both).

### Effect of Raw Lacquer Extract and ONC201 Co-treatment on TRAIL, DR5, and Cleaved Caspase 8 Expression

**Figure 4.**
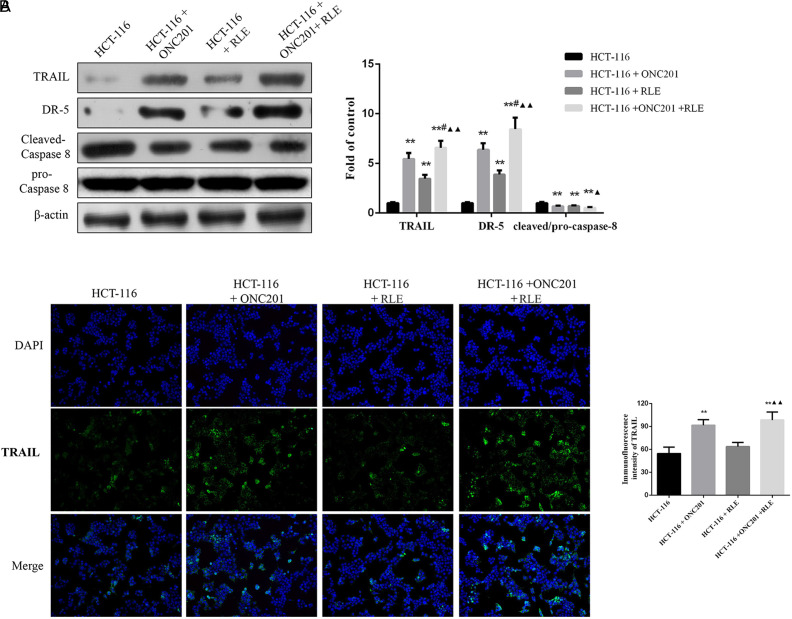
Effect of RLE and ONC201 co-treatment on TRAIL, DR5, cleaved caspase 8, and pro-caspase 8 expression levels. (A) Western blot analysis for the detection of protein expression of TRAIL, DR5, cleaved caspase 8, and pro-caspase 8. (B) Immunofluorescence expression of TRAIL in HCT116 cells. ^*^ *P* < .05, ^**^ *P* < .01 vs. the HCT116 group; ^#^ *P* < .05, ^##^ *P* < .01 versus the HCT116 + ONC201 group; ^▲^ *P* < .05, ^▲▲^ *P* < .01 vs. the HCT116 + RLE group. RLE, raw lacquer extract.

The expression levels of TRAIL were further explored using western blotting and immunofluorescence assays. As shown in Figure 4A, the expression degree of TRAIL protein in HCT116 cells treated with ONC201 (10 μM), RLE (0.5 mg/mL), or co-treatment was raised notably in comparison to the control HCT116 cells (all *P *< .01). Furthermore, TRAIL protein expression level in co-treatment group cells was the highest. Similar effects of RLE (0.5 mg/mL) and ONC201 (10 μM) co-processing on the expression degree of DR5 protein in HCT116 cells were also found. While for cleaved caspase 8, the results showed that HCT116 cells treated with ONC201 (10 μM), RLE (0.5 mg/mL), or co-treatment significantly inhibited the cleaved caspase 8 protein expression levels than that in the control HCT116 cells (*P *< .01). ONC201 (10 μM) and RLE (0.5 mg/mL) co-treatment further enhanced the suppression effect on the cleaved caspase 8 protein expression than that in the HCT116+RLE group (*P *< .05). Also, immunofluorescence assays found a significantly higher intensity of green fluorescence TRAIL staining in the ONC201 (10 μM), as well as ONC201 (10 μM) and RLE (0.5 mg/mL) co-treatment groups in contrast to the control HCT116 group (*P *< .01, Figure 4B).

### Effect of RLE and ONC201 Co-Processing on p-mTOR/mTOR and p-S6K/S6K Proteins Degrees in HCT116 Cells

**Figure 5.**
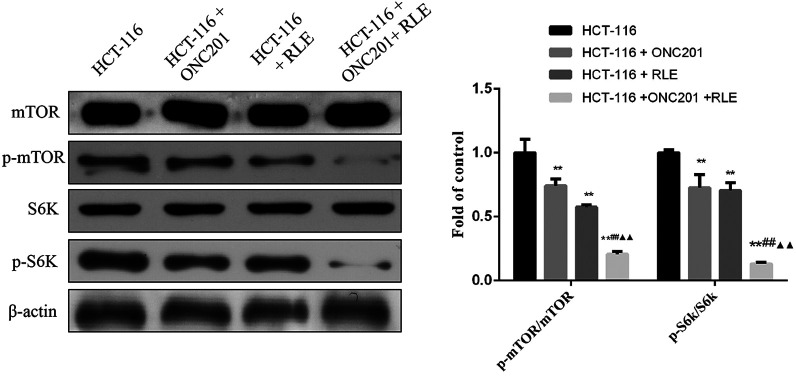
Effect of RLE and ONC201 co-treatment on p-mTOR/mTOR and p-S6K/S6K protein expression levels in HCT116 cells. ^*^ *P* < .05, ^**^ *P* < .01 vs. the HCT116 group; ^#^ *P* < .05, ^##^ *P* < .01 vs. the HCT116 + ONC201 group; ^▲^ *P* < .05, ^▲▲^ *P* < .01 vs. the HCT116 + RLE group. RLE, raw lacquer extract.

The possible inhibition mechanism of RLE-mediated sensitization of ONC201 to HCT116 cells was further investigated. Western blot analysis showed that ONC201 (10 μM), RLE (0.5 mg/mL), and ONC201 (10 μM) and RLE (0.5 mg/mL) co-treatment inhibited the degrees of p-mTOR/mTOR and p-S6K/S6K proteins significantly *(P *< .01) (Figure 5). ONC201 (10 μM) and RLE (0.5 mg/mL) co-treatment enhanced the inhibitory effect on the expression of p-mTOR/mTOR and p-S6K/S6K protein significantly in contrast to the individual treatment groups (all *P *< .01).

### Effect of RLE and ONC201 Co-Treatment on Bcl-2 and Bax Expression Levels in HCT116 Cells

**Figure 6.**
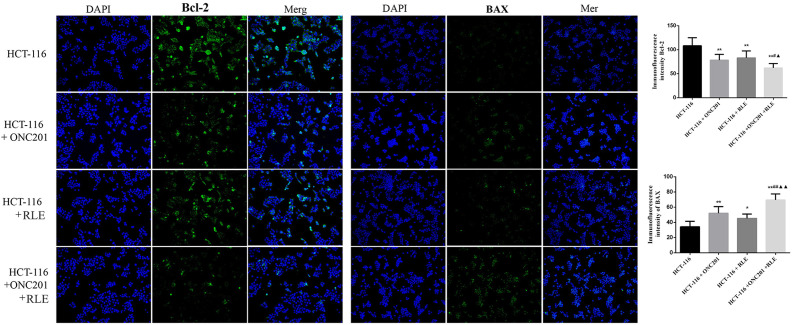
Immunofluorescence assays for assessing the effects of RLE and ONC201 co-treatment on Bcl-2 and Bax expression in HCT116 cells. Magnification ×200. ^*^ *P* < .05, ^**^ *P* < .01 vs. the HCT116 group; ^#^ *P* < .05, ^##^ *P* < .01 vs. the HCT116 + ONC201 group; ^▲^ *P* < .05, ^▲▲^ *P* < .01 vs. the HCT116 + RLE group. RLE, raw lacquer extract.

The expression of apoptosis-related proteins, Bax, and Bcl-2, was also investigated through immunofluorescence assays (Figure 6). The results demonstrated that the fluorescence intensity of Bcl-2 in HCT116 cells treated with ONC201 (10 μM), RLE (0.5 mg/mL), or co-treatment was decreased in contrast to the control group in HCT-116 cells, and the fluorescence intensity of Bcl-2 in the co-processing group was also immensely lower than the intensity in ONC201 or RLE individually treated groups (*P *< .05 for both). However, the fluorescence intensity of Bax in the HCT116 cells treated with ONC201 (10 μM), RLE (0.5 mg/mL), or ONC201 (10 μM) and RLE (0.5 mg/mL) co-treatment were all increased significantly compared with the control cells, and the fluorescence intensity in the co-processing group was immensely higher than the intensity in ONC201 (10 μM) or RLE (0.5 mg/mL) treated alone (*P *< .01 for both).

## Discussion

For both sexes, CRC is the third most generally diagnosed cancer (10.2% of the total cancer events) and the second lethal cause of cancer (9.2% of the total death cancer cases) worldwide.^1^ According to recent estimates, for all stages of CRC, the 5-year relative survival is 65%, however, this rate decays to 12% for stage IV.^21^ Along with the implementation of the nationwide regular CRC screening program, developing therapeutic approaches is also urgent for reducing CRC morbidity and mortality. In the present study, ONC201 and RLE treatment demonstrated effective anti-cancer activities against CRC HCT116 cells. Notably, RLE and ONC201 co-treatment enhanced the anti-CRC cell activity of HCT116 cells. Furthermore, ONC201 induced TRAIL/DR-5 and caspase-8 protein expression as well as reduced mTOR phosphorylation, and this effect was also potentiated with RLE co-treatment to some extent.

*Toxicodendron vernicifluum* has an age-old tradition of medicinal history in Asia for the treatment of various diseases, including cancer.^8^ In advanced non-small cell lung cancer patients following the preliminary round of chemotherapy, a study showed that allergen-removed Rhus verniciflua Stokes extract encourages enhanced progression-free survival and overall survival in contrast to those in historical controls.^22^ In cancer cells, the ethanol extract of *T. vernicifluum* induces cell apoptosis through the induction of caspase-3, 8, 9 and Bax expression, as well as the restraint of Bcl-2 protein level, the secretion of cytochrome C, and the regulation of cell cycle progression.^23,24^ The fisetin from *T. vernicifluum* is reported to restrain cell viability and lead to apoptosis of chemoresistant CRC cells and inhibit tumor growth in the xenograft model too.^25^ Raw lacquer extract is the major component of *T. vernicifluum*. In this article, except for the observed inhibition impact of ONC-201 in HCT116 cells, we found that administration of RLE into HCT116 cells showed anti-cancer activities on CRC cells. The MTT, trypan blue staining, colony formation, wound healing, and transwell assays showed that HCT116 cell’s viability, survival, proliferation, migration, and invasion were all inhibited by RLE treatment. Raw lacquer extract was further found to induce cell apoptosis and led to an advance in the expression degrees of apoptosis-related proteins, containing cleaved caspase 8 and Bax, as well as correspondingly reduce the expression levels of Bcl-2. These results indicated that RLE exhibits anti-cancer activity in HCT116 cells through the regulation of cell activities.

ONC201 is a p-53-dependent inducer of TRAIL transcription. It was a novel class of anti-cancer agents and is being tested in phase II clinical trials at the present time.^6^ Other studies report that ONC201 induces tumor cell death through TRAIL-dependent mechanisms.^7,26^ ONC201 has anti-tumorigenic and anti-­metastatic activities in uterine serous carcinomas through the TRAIL-mediated apoptotic pathway and the inactivation of the AKT/MAPK signaling pathways.^18^ Prabhu et al^7^ also reported that ONC201/TIC10 is an effective combination for CRC treatment. It targets CRC cancer stem cells (CSCs) by impacting Akt-Foxo3a-TRAIL to suppress CSCs self-renewal and restrain CSC-initiated xenograft tumor development. In the current study, the results showed that ONC201 demonstrated effective anti-CRC cell activity in HCT116 cells. HCT116 cell viability, growth, migration, and invasion were significantly inhibited by ONC201 treatment. ONC201 also exerted cytotoxicity and apoptosis effects on HCT116 cells, and these anti-tumor effects appeared to be acted through the regulation of TRAIL/DR-5L and mTOR expression in CRC cells.

The mTOR signaling pathway is frequently involved in a wide spectrum of cellular functions, including cell proliferation, autophagy, and metabolism. The crucial role of mTOR in various types of human cancers has also raised interest in the development of mTOR inhibitors for new therapies.^27,28^ In a previous study, the TRAIL inducer, ONC201, exhibited anti-CRC cell activity and mTOR inhibition effect in CRC cells, and ONC201-induced cell cytotoxicity, and apoptosis were also potentiated by co-treatment with mTOR inhibitors, AZD-8055, by blocking mTOR and Akt activation.^8^ This provides a research direction for inhibiting the drug resistance of advanced cancer cells. The active compound of fisetin in RLE has been found to induce inhibition of the mTOR signaling pathway, resulting in restraint of translation of prostate cancer-dependent and initiation of autophagy in PC3 cells.^29^ Consequently, in this article, it was assumed that RLE has an anti-cancer effect against CRC cells, and it may have a synergistic effect with ONC201 against CRC. The results showed that RLE treatment to CRC cells inhibited cell viability and induced cell apoptosis. RLE and ONC201 co-treatment enhanced the anti-CRC cell activity in HCT116 cells. Notably, mTOR and S6K phosphorylation were both inhibited following RLE treatment, and this inhibition was potentiated with ONC201 co-treatment. This result indicated that mTOR may be involved in the anti-CRC effects of RLE and ONC201, and restraint of mTOR by RLE may bring about sensitizing the ONC201-induced anti-CRC cell survival.

In summary, this article investigated the therapeutic effect of RLE in CRC cells and explored the probable synergistic function of RLE on ONC201-inspired anti-cancer activity. The results showed that ONC201 and RLE demonstrated effective anti-CRC cell activity in HCT116 cells. The treatment inhibited the viability of HCT116 cells and proliferation and inspired apoptosis in HCT116 cells. The HCT116 cell migratory and invasive abilities were also inhibited by ONC201 and RLE administration. Significantly, RLE and ONC201 co-treatment enhanced the anti-CRC cell activity in HCT116 cells. ONC201 raised TRAIL/DR-5 protein expression degrees and reduced p-mTOR/mTOR and p-S6K/S6K phosphorylation, the effect that was also potentiated with RLE co-treatment. This research proposed that RLE could be served as an adjuvant agent in combination with ONC201 to enhance the inhibitory effects on CRC HCT116 cell activity. This article supplied a possibility for the clinical application of ONC201 and RLE co-therapy and provides an experimental basis for the discovery of drugs for the intervention of CRC.
